# Correction: Yin et al. Improvement of SAM2 Algorithm Based on Kalman Filtering for Long-Term Video Object Segmentation. *Sensors* 2025, *25*, 4199

**DOI:** 10.3390/s25165131

**Published:** 2025-08-19

**Authors:** Jun Yin, Fei Wu, Hao Su, Peng Huang, Yuetong Qixuan

**Affiliations:** 1 School of Computer Science and Technology, Zhejiang University, Hangzhou 310058, China; 12221242@zju.edu.cn (J.Y.); wufei@zju.edu.cn (F.W.); 2 Zhejiang Dahua Technology Co., Ltd., Hangzhou 310053, China; huang_peng@dahuatech.com (P.H.); qi_xuanyuetong@dahuatech.com (Y.Q.)

In the original publication [1], there were some errors in the main text. The corrected content appears below.

1. Introduction

Paragraph 2: “visual object tracking (VOT)” is replaced by “VOS”.

Paragraph 3: “VOT” is replaced by “visual object tracking (VOT)”.

Paragraph 4: “tracking performance in VOT scenarios.” is replaced by “segmentation performance in VOS scenarios.”

Paragraph 6: ‘tracking’ is replaced by a ‘segmentation’.

3.2. Kalman Filtering-IoU Fusion Framework

Paragraph 1: “To improve tracking consistency, we propose the Kalman Filtering-IoU (KF-IoU) fusion framework, which introduces a predictive component to stabilize tracking and reduce the impact of single-frame errors.” is replaced by a “Given the success of SAMURAI [5] in the VOT task, to improve tracking consistency in VOS scenarios, we propose the Kalman Filtering-IoU (KF-IoU) fusion framework, which introduces a predictive component via Kalman Filtering to stabilize tracking and reduce the impact of single-frame errors while ensuring spatial segmentation precision through IoU optimization.”

Paragraph 6: Add “Following [5], the KF-IoU fusion function is defined as follows:”

3.3. Adaptive Historical Frame Selection Strategy Based on Dynamic Threshold

Paragraph 4: “The validity of the historical frame *f_i_* is determined by the following conditions simultaneously:” is replaced by “The validity of the historical frame *f_i_* is determined by the following conditions simultaneously [5]:”

6. Conclusions

Paragraph 1: ‘tracking’ is replaced by a ‘segmentation’.

The authors state that the scientific conclusions are unaffected. This correction was approved by the Academic Editor. The original publication has also been updated.
